# The Good and the Bad of SHROOM3 in Kidney Development and Disease: A Narrative Review

**DOI:** 10.1177/20543581231212038

**Published:** 2023-12-13

**Authors:** Amy Paul, Allison Lawlor, Kristina Cunanan, Pukhraj S. Gaheer, Aditya Kalra, Melody Napoleone, Matthew B. Lanktree, Darren Bridgewater

**Affiliations:** 1 Department of Pathology and Molecular Medicine, Faculty of Health Sciences, McMaster University, Hamilton, ON, Canada; 2 Department of Health Research Methods, Evidence, and Impact, Faculty of Health Sciences, McMaster University, Hamilton, ON, Canada; 3 Population Health Research Institute, Hamilton, ON, Canada; 4 Division of Nephrology, Department of Medicine, McMaster University, Hamilton, ON, Canada

**Keywords:** SHROOM3, genome-wide association studies (GWAS), chronic kidney disease (CKD), kidney development, epithelial morphology

## Abstract

**Purpose of review:**

Multiple large-scale genome-wide association meta-analyses studies have reliably identified an association between genetic variants within the *SHROOM3* gene and chronic kidney disease. This association extends to alterations in known markers of kidney disease including baseline estimated glomerular filtration rate, urinary albumin-to-creatinine ratio, and blood urea nitrogen. Yet, an understanding of the molecular mechanisms behind the association of SHROOM3 and kidney disease remains poorly communicated. We conducted a narrative review to summarize the current state of literature regarding the genetic and molecular relationships between SHROOM3 and kidney development and disease.

**Sources of information:**

PubMed, PubMed Central, SCOPUS, and Web of Science databases, as well as review of references from relevant studies and independent Google Scholar searches to fill gaps in knowledge.

**Methods:**

A comprehensive narrative review was conducted to explore the molecular mechanisms underlying SHROOM3 and kidney development, function, and disease.

**Key findings:**

SHROOM3 is a unique protein, as it is the only member of the SHROOM group of proteins that regulates actin dynamics through apical constriction and apicobasal cell elongation. It holds a dichotomous role in the kidney, as subtle alterations in SHROOM3 expression and function can be both pathological and protective toward kidney disease. Genome-wide association studies have identified genetic variants near the transcription start site of the *SHROOM3* gene associated with chronic kidney disease. SHROOM3 also appears to protect the glomerular structure and function in conditions such as focal segmental glomerulosclerosis. However, little is known about the exact mechanisms by which this protection occurs, which is why SHROOM3 binding partners remain an opportunity for further investigation.

**Limitations:**

Our search was limited to English articles. No structured assessment of study quality was performed, and selection bias of included articles may have occurred. As we discuss future directions and opportunities, this narrative review reflects the academic views of the authors.

## Abrégé

**Contexte motivant la revue:**

Plusieurs méta-analyses d’envergure portant sur des études d’association pangénomiques ont permis d’identifier de manière fiable une association entre des variants génétiques du gène SHROOM3 et l’insuffisance rénale chronique. Cette association s’étend aux altérations des marqueurs connus de l’insuffisance rénale, notamment le débit de filtration glomérulaire estimé initial, le rapport albumine/créatinine urinaire et le taux d’urée dans le sang. Pourtant, la compréhension des mécanismes moléculaires qui sous-tendent cette association entre SHROOM3 et l’insuffisance rénale reste mal communiquée. Nous avons procédé à une revue narrative afin de résumer l’état actuel de la littérature en ce qui concerne les relations génétiques et moléculaires entre SHROOM3 et le développement des reins et de l’insuffisance rénale.

**Sources:**

Les bases de données PubMed, PubMed Central, SCOPUS et Web of Science. L’examen des références des études pertinentes et des recherches indépendantes sur Google Scholar a également été réalisé pour combler les lacunes dans les connaissances.

**Méthodologie:**

Une revue narrative complète a été effectuée afin d’explorer les mécanismes moléculaires qui sous-tendent SHROOM3, le développement des reins, la fonction rénale et l’insuffisance rénale.

**Principaux résultats:**

SHROOM3 est une protéine unique puisqu’elle est la seule du groupe de protéines SHROOM à réguler la dynamique de l’actine par la constriction apicale et l’élongation des cellules apico-basales. SHROOM3 joue un rôle dichotomique dans le rein; de subtiles altérations de son expression et de sa fonction pouvant à la fois être pathologiques ou protectrices en contexte d’insuffisance rénale. Des études d’association pangénomiques ont permis d’identifier des variants génétiques associés à l’insuffisance rénale chronique près du site d’initiation de la transcription du gène SHROOM3. SHROOM3 semble également protéger la structure et la fonction des glomérules dans des contextes comme la glomérulosclérose segmentaire focale. On en sait toutefois peu sur les mécanismes précis qui entraînent cette protection; les partenaires de liaison de SHROOM3 demeurent par conséquent d’intéressantes avenues pour une étude plus approfondie.

**Limites:**

Notre recherche était limitée aux articles rédigés en anglais. Les études pertinentes n’ont pas fait l’objet d’une évaluation structurée de leur qualité. Un biais de sélection des articles inclus peut s’être produit. Bien que nous discutions des orientations et des possibilités futures, cette revue narrative reflète les points de vue académiques des auteurs.

## Introduction

Kidney disease is a global health concern affecting over 10% of the adult population worldwide and approximately 3 million Canadians.^1,2^ Inherited and acquired conditions including diabetes, hypertension, glomerular disease, infections, and abnormal development may reduce nephron number, resulting in a decline of estimated glomerular filtration rate (eGFR), metabolic and systemic changes including hypertension, edema, uremia, and progression to chronic kidney disease (CKD).^3,4^ SHROOM3 is an actin-binding protein that regulates epithelial cell shape during development.^5-8^ First identified through investigations of neural tube defects (NTDs) in embryonic mice, “Shroom” was named after its mutant phenotype that demonstrated neural folds “mushrooming” away from the dorsal midline.^6^ Over the past 15 years, human population-scale genome-wide association studies (GWAS) found significant association of genetic variants in *SHROOM3* with eGFR, urinary albumin-to-creatinine ratio (uACR), blood urea nitrogen (BUN), and stage 3 CKD.^9-13^ Many follow-up GWAS have evaluated patient populations with different ancestries and replicated the associations between *SHROOM3* genetic variants and CKD.^10-16^ This review focuses on the experimental evidence that confirms the important roles for SHROOM3 in kidney development, function, and disease.

## Methods

A comprehensive narrative review was preformed to explore perspectives of SHROOM3 and kidney development and disease, including cell, mouse model, and genomic studies. We included English peer-reviewed primary research articles examining SHROOM3 and excluded conference and meeting abstracts. PubMed, PubMed Central, SCOPUS, and Web of Science databases were searched using search terms “Shroom3 OR Shrm3,” “Kidney development,” “Chronic kidney disease OR CKD,” and “Kidney disease.” Our first round of screening involved scanning the title and abstracts of all search results and categorizing them based on relevancy and predefined inclusion and exclusion criteria. All primary authors reviewed relevant articles and performed a second round of screening including full-text analysis to confirm the category and relevancy of articles and establish a consensus on the articles to be included in the review. We critically examined existing literature and looked for common themes through discussion. A review of the referenced literature in the included studies was also completed and relevant sources were added to our narrative review (**Supplementary Figure 1**).

## Review

### *SHROOM3* Gene and Protein Structure

**Figure 1.**
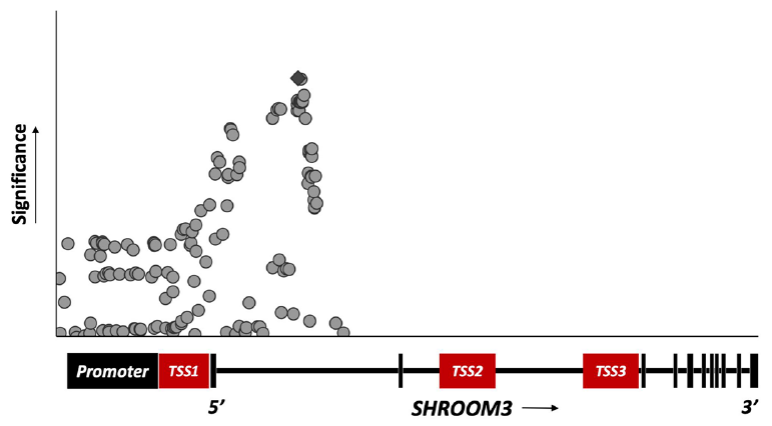
Significant associations between genetic variants in *SHROOM3* and chronic kidney disease. *Note.* Each dot represents a genetic variant positioned on the X-axis based on its physical location on the gene. The Y-axis reflects the level of significance regarding its association with chronic kidney disease calculated as −log(*P* value). Only the 5’ region of *SHROOM3* contains significantly (*P* value < 5 × 10^−8^) associated variants with chronic kidney disease. The transcription start sites (TSSs), TSS1 and TSS2/3, encode the SHROOM3 long isoform and short isoform, respectively.

**Figure 2.**
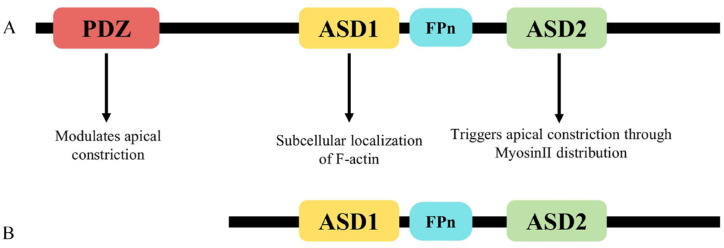
*SHROOM3* gene and protein structure. *Note.* (A) Schematic representing the long isoform consisting of a central ASD1 domain, C-terminal ASD2 domain, proline-rich (FPn) domain, and N-terminal PDZ domain. This isoform is made up of 1986 amino acids, which is around 220 kilodaltons (KD) in size. (B) Schematic representing the short isoform consisting only of the ASD1, ASD2 domain, and proline-rich (FPn) domain. This isoform lacks the PDZ domain and therefore consists of 1808 amino acids, which is around 200 KD in size. ASD = apical protein *Xenopus*/SHROOM domain.

The human SHROOM3 protein is encoded by the *SHROOM3* gene, which is made up of 348 kilobase pairs located on the long arm of chromosome 4.^17^ Among the 17 exons found in *SHROOM3*, the most common mRNA transcript (canonical transcript) includes only 11 of the 17 exons due to alternative transcriptional start sites (TSSs) (**Figure 1**).^18^ These 11 exons produce a 5991 base pair transcript resulting in a 1986 amino acid protein that is 220 kilodaltons (**Figure 2A**).^17^ *SHROOM3* has 3 transcription start sites—TSS1 encodes the long SHROOM3 isoform containing all 11 exons whereas TSS2 and TSS3 both encode a shorter isoform containing exons 3 through 11 (**Figure 1**).^18^ Although 2 “long” and “short” SHROOM3 isoforms are most common, 13 different *SHROOM3* transcripts originating from the 3 TSSs have been identified from RNA-sequencing experiments.^18,19^ The long isoform consists of a central apical protein *Xenopus*/SHROOM domain 1 (ASD1) that interacts directly with F-actin, a C-terminal ASD2 domain that triggers apical constriction and reorganization of the actomyosin network, and an N-terminal PDZ domain responsible for mediating protein-protein interactions that facilitate cell signaling (**Figure 2**).^6,7,20^ The short isoform contains the ASD1 and ASD2 domains but lacks the PDZ domain. An investigation into SHROOM3 isoforms and renal functionality revealed that the short isoform exhibits functional capacity despite lacking the PDZ domain, yet experimental isoforms with absent ASD2 domains demonstrate no functional capacity, indicating that the ASD2 domain is vital for protein function.^18^ Conditions that alter SHROOM3 expression over time and between cell types remain unknown. However, based on the different protein domains, it is likely that the different isoforms play unique roles in various cells that could address its role in CKD.

### SHROOM3 Mechanisms of Action

**Figure 3.**
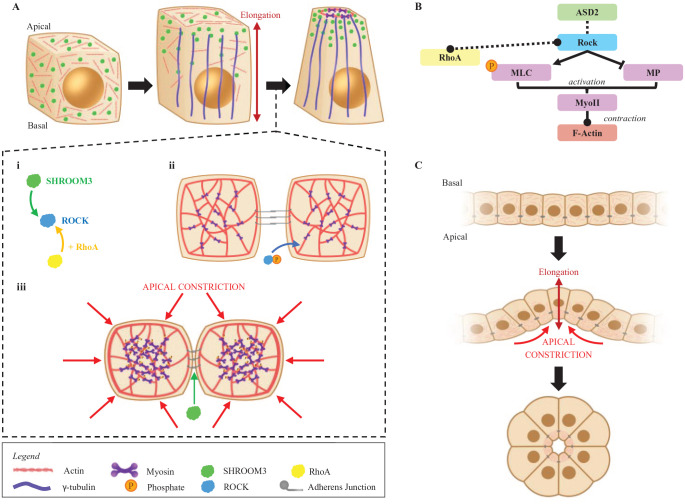
SHROOM3 in apical constriction and apicobasal cell elongation. *Note.* (A) SHROOM3 organizes F-actin to the apical surface of cuboidal cells. Simultaneously, γ-tubulin apically accumulates to facilitate cell elongation along the apicobasal axis. (Ai-iii) A close-up, birds-eye view of the apical surface of the cell during the intermediate step of apical constriction. SHROOM3 binds and recruits actin and ROCK proteins to apical junctions in cells. Once ROCKs are activated by RhoA, they phosphorylate myosin, resulting in a contractile force that regulates actomyosin dynamics. (B) A schematic presentation of the RhoA-dependent actomyosin activation pathway. (C) Broad overview of a sheet of cells undergoing cell elongation and apical constriction to form tubules. ROCK = Rho-associated kinases; RhoA = Ras homolog family member A; MLC = myosin regulatory light chain; MP = myosin phosphatase.

The involvement of Shroom3 in neural tube closure, as seen in mice and *Xenopus*, provided insight into SHROOM3’s role in cell shape changes during tissue morphogenesis.^6^ Although SHROOM proteins 1 through 3 can coordinate the assembly of microtubules and actin cytoskeletons, SHROOM3 is unique, as it is the only member from the SHROOM family of proteins capable of eliciting significant actin recruitment to facilitate apical constriction.^5,21^ SHROOM3 affects epithelial cell shape by modulating the actin cytoskeleton, which can initiate apical constriction and apicobasal cell elongation (**Figure 3**).^6,7,20,22,23^ Apical constriction occurs when polarized epithelial cells take on a wedge shape during the morphogenesis of epithelial sheets.^6^ To initiate this process, SHROOM3 recruits and directly binds to F-actin at the sites of adherens junction complexes. This SHROOM3-actin complex also binds to myosin, resulting in the contraction of circumferential actin belts and subsequently, apical constriction.^24^ Specifically, it is the ASD1 domain within SHROOM3 that binds to F-actin and regulates its subcellular localization.^6,7,20^ The ASD2 domain binds directly to Rho-associated kinases (ROCK)—ROCK1 and ROCK2.^8,25,26^ Ras homolog family member A (RhoA) is activated by Trio, a RhoA guanine nucleotide exchange factor, to activate the ROCK proteins.^8,25,26^ The ROCK proteins then phosphorylate myosin regulatory light chain (MLC) to activate myosin II, resulting in F-actin contraction.^8,27-30^ This molecular pathway is supported by studies demonstrating that SHROOM3 colocalizes with actin filaments, RhoA, ROCKs, and myosin II at adherens junctions (**Figure 3A-C**).^8,31-34^ In connection to the kidneys, the absence of apical ROCK1 expression and phosphorylation was observed in *Shroom3* homozygous null mutant mice podocytes, which further confirmed that SHROOM3 regulates the actomyosin cytoskeleton through the ROCK/MyoII signaling pathways.^35^

Studies have also defined a role for SHROOM3 in regulating apicobasal cell elongation, a process by which epithelial sheets thicken during cell shape changes.^22,23^ In epithelial cells, microtubules are aligned with the apicobasal axis and assembled by microtubule-organizing centers, namely, γ-tubulin.^21,36^ In the absence of SHROOM3 expression, γ-tubulin fails to accumulate in the apical regions of the cells, resulting in reduced apicobasal cell elongation.^21^ Altogether, these studies demonstrate that SHROOM3 modulates the accumulation of actomyosin to induce apical constriction and γ-tubulin to induce apicobasal cell elongation during epithelial morphogenesis.^7,8,21,37^ These processes are essential to key developing organ epithelia, including the kidneys.^38,39^

### SHROOM3 Expression in the Developing and Adult Kidney

#### Kidney Development

**Figure 4.**
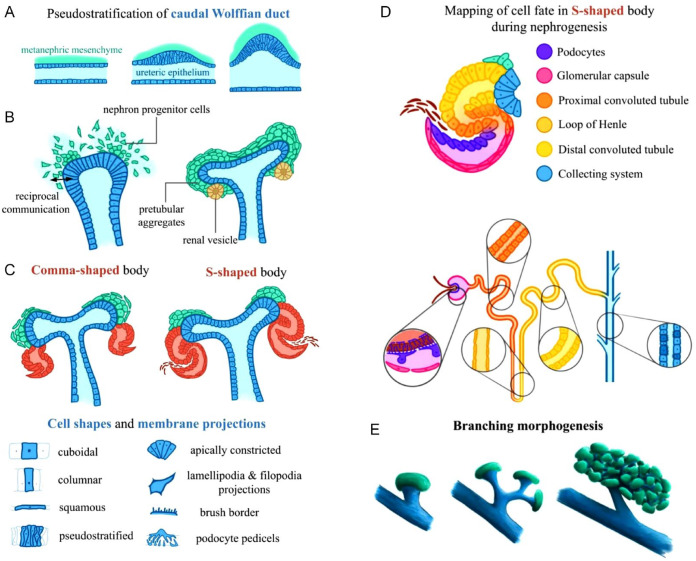
Kidney epithelial morphogenesis during development. *Note.* (A) Wolffian duct transitions from cuboidal cells to a pseudostratified epithelium to form the ureteric bud (dark blue). (B) Ureteric epithelium signals the nephron progenitor cells (green) to proliferate, migrate, condense, and change shape around the ureteric tip to eventually form peritubular aggregates. (C) As a result of significant epithelial morphogenesis, peritubular aggregates (red) transition from the comma-shaped to S-shaped body stage. (D) Cell fate mapping of S-shaped body (see nephron legend) forming a mature nephron. (E) Nephron progenitor cells signal the ureteric epithelial tip to go through multiple rounds of budding, elongation, and branching.

During kidney development, many signaling pathways regulate epithelial morphogenesis.^40^ This process is initiated when the Wolffian duct transitions from a cuboidal to pseudostratified epithelium to mediate the initial outgrowth of the ureteric bud from the duct (**Figure 4A**).^41,42^ This starts branching morphogenesis which forms the renal collecting duct system. In a reciprocal fashion, the nephron progenitor cells receive signals that instruct the cells to accumulate around the tips of the ureteric bud. During this process, the nephron progenitor cells change their cell shape and develop membrane specializations including filopodia and lamellipodia prior to forming pretubular aggregates (**Figure 4B**).^43,44^ Pretubular aggregates rearrange and transition into spherical renal vesicles. These vesicles move through a series of morphological stages including the comma-shaped body and S-shaped body (**Figure 4C**).^45-47^ At the final stage, cells of the developing nephrons will take on cell shapes like columnar, cuboidal, or squamous depending on specific nephron segments. Some also form function-specific structures, such as podocyte pedicels and the proximal tubule brush border membrane. This process culminates in the formation of a mature nephron, the functional unit of the kidney (**Figure 4D**).^48-50^

#### SHROOM3 Expression Pattern

*Shroom3* mRNA expression was detected through *in situ* hybridization in the pronephric kidney of *Xenopus*, demonstrating the potential that its function in the kidney is conserved.^5^ To further characterize expression, single-cell RNA-sequencing (scRNAseq) defined *SHROOM3* mRNA expression from 17-week-old human embryonic kidneys in most kidney cell types, with the highest expression detected in the nephron progenitor cells.^51,52^ Future RNAseq investigations will be helpful in characterizing embryonic *Shroom3* mRNA expression in mouse kidney cells. In adult human kidneys, RNAseq data from the Genotype-Tissue Expression (GTEx) project revealed *SHROOM3* mRNA expression in both the kidney cortex and medulla.^17^ *SHROOM3* mRNA expression was found to be the highest in type A intercalated cells and distal convoluted tubule cells.^53^ Adult wild-type mice scRNAseq showed that Shroom3 expression is most prominent in the podocytes, ascending limb loop of Henle cells, and mesangial cells.^54^

Although the RNAseq data is informative, defining the specific spatial and temporal expression pattern is an important step in verifying these large data sets. This was addressed by introducing a *LacZ* reporter gene in the original gene trap strategy allowing for the analysis of endogenous *Shroom3* gene expression.^6^ In the developing embryonic kidney, LacZ staining was observed in the nephron progenitors, the renal vesicle, and the developing parietal and visceral epithelial cell layers. Expression was also observed in the tips of the ureteric epithelium, distal collecting ducts, and the proximal renal pelvis. *In situ* hybridization and immunohistochemistry confirmed similar mRNA and protein expression patterns of Shroom3. However, the immunohistochemistry studies demonstrated not all nephron progenitors, ureteric tips, and collecting ducts cells express the Shroom3 protein. This could be due to poor specificity of antibodies, low protein expression in some cells, or differences in Shroom3 isoforms.^35^

In the postnatal kidney, *Shroom3* expression was observed in glomeruli, and in some medullary and cortical tubules.^35^ Within the glomerulus, LacZ staining was observed in the parietal epithelium and visceral podocyte epithelium, whereas no expression was observed in the mesangial or endothelial cells. LacZ expression was also demonstrated in select tubular epithelial, primarily in the proximal tubules.^35,55^ Additional studies confirm consistency in protein expression in some, but not all, epithelial cells in the apical regions of the initial S1 segment of the proximal tubule, distal convoluted tubule, and medullary collecting ducts.^56,57^ Studies have also demonstrated *Shroom3* mRNA expression in the pronephric tubules in zebrafish, the pronephric duct in *Xenopus*, and in the glomeruli of both.^5,58^ RNA-sequencing in human adult tissue showed spatial expression of SHROOM3 transcripts in kidney glomeruli and tubules.^18^ As SHROOM3 is a driver of cell morphology, these expression patterns suggest important roles for SHROOM3 in kidney function and maintenance of kidney tissue. To date, no studies have defined the temporal protein expression in human adult tissue, and the temporal and spatial protein expression has yet to be defined in human embryonic tissue. Altogether, the expression of SHROOM3 is dynamic and plays distinct functions in specific cells through different stages of development.

### SHROOM3 in Human Kidney Disease

Genome-wide association studies identified that common genetic variants near the *SHROOM3* gene are associated with eGFR and prevalent CKD with modest effect sizes.^10,11^ Plotting these GWAS show that the associated genetic variants localize around TSS1 and the 5’ end of *SHROOM3* (**Figure 1**).^18^ Given their proximity to the transcription start site, it is predicted that the associated genetic variants could decrease or increase SHROOM3 expression. However, the most studied variant is the intronic *SHROOM3* rs17319721 (Risk allele frequency in general population: 35%; forward strand risk allele: A, other allele: G).^10,11,59^ The allele frequency of this variant is high in the general population likely because its effects on kidney health and function are not as severe. Highly pathogenic variants would be represented as a much lower frequency due to the more severe effects on health. In a study of 540 3-month post-transplant kidney biopsies, for each rs17319721 risk allele in the donor kidney, there were greater *SHROOM3* mRNA expression (*P* < .05) and increased risk of chronic allograft nephropathy in the transplant recipients (odds ratio = 1.98, confidence interval = 1.10-3.59). The association with chronic allograft nephropathy is thought to be due to the rs17319721 variant generating a T-cell factor/lymphoid enhancer factor (TCF/LEF) transcription factor binding site in the intronic region between exons 1 and 2 of the *SHROOM3* gene. This binding site results in the formation of a complex between TCF7L2 and transcriptional activator β-catenin, which increases *SHROOM3* gene transcription. *SHROOM3* overexpression amplifies the TGFβ1/SMAD3 signaling pathway, which upregulates profibrotic pathways leading to allograft fibrosis.^59^ These observations highlight how *SHROOM3* overexpression has a significant impact on profibrotic signaling pathways, which may cause poor kidney health outcomes.

In addition, higher *SHROOM3* mRNA expression as a result of rs17319721 was found to be associated with reduced cross-sectional eGFR and reduced uACR. This association is considered to be contradictory as typically, most kidney diseases manifest with reduced eGFR and/or increased uACR. An association with kidney disease was still present despite these kidney function measures displaying the same direction of association. A possible explanation for this is the state of hyperfiltration caused by conditions such as diabetes, sickle cell disease, hyperuricemia, hypertension, or primary aldosteronism. These diseases are often characterized by a period of increased eGFR and the delayed onset of elevated uACR which can eventually progress to CKD.^9^ It is possible that a similar phenomenon can explain how same direction associations due to SHROOM3 overexpression can still give rise to kidney disease symptoms.

Rare *SHROOM3* coding variants also appear to support a link between *SHROOM3* function and kidney disease.^18^ The highly conserved *SHROOM3* missense variant, rs181194611 (Forward Strand; Risk allele frequency in general population: 0.02%; Risk allele: T, Other allele C) is located at the binding site for the protein named 14-3-3, which regulates the Hippo signaling pathway. The Hippo signaling pathway regulates cell proliferation and differentiation, yet when disrupted, it is known as a central player in several kidney diseases.^60^ Rare variants such as rs181194611 have the power to disrupt normal SHROOM3 protein function and cause major disturbances in normal signaling pathways which could lead to alterations in kidney health outcomes.^60-62^

### Shroom3 in Animal Models of Kidney Disease

The complete knockout of *Shroom3* has been performed by inserting a cassette that contains a premature stop codon between exons 3 and 4. The cassette prevents the transcription and translation of key Shroom3 domains, resulting in nonsense-mediated mRNA decay which prevents Shroom3 protein production.^6^ The analysis of homozygous *Shroom3* null mice kidneys in the early embryonic period (E13.5 and E16.5) demonstrated slightly smaller kidneys, but no significant developmental abnormalities in branching morphogenesis at E13.5 and E16.5. However, the developing nephrons in null mutants exhibited collapsing glomeruli and early nephron loss as indicated with the reduction in nephron number at E18.5.^35^ The remaining homozygous *Shroom3* null glomeruli at E18.5 exhibited small podocyte cell bodies with short and disorganized foot process interdigitation and disrupted expression of proteins essential for maintenance of the glomerular filtration barrier. Heterozygous *Shroom3* null mice display a similar, yet less severe, embryonic phenotype.^35,55^ Postnatally, apart from rare instances of a unilateral hypoplastic kidney at 1 month, the gross and histological analysis of heterozygous *Shroom3* null mice kidneys appears normal. At the molecular level, some alterations in the apical-basolateral orientation of tubular epithelium were observed at 3 months, but there were no alterations in kidney function or blood pressure.^56^ Unfortunately, homozygous *Shroom3* null mice die perinatally due to failure in neural tube closure, precluding postnatal analysis.^6,63^ These studies highlight an important role for *Shroom3* in kidney development that could manifest in kidney disease under specific conditions.

Evidence that reduced Shroom3 expression could contribute to kidney disease postnatally was gathered when 3-month-old *Shroom3* heterozygous null mice were exposed to an acute kidney injury. The *Shroom3* heterozygous null mice revealed an increased sensitivity to kidney injury, specifically worse tubular injury 48 hours after injury that failed to recover after 10 days. This altered recovery was due to disrupted Rho-kinase signaling and actin disorganization that resulted in disrupted epithelial differentiation of the tubular epithelium.^57^ The changes in Shroom3 expression in heterozygous null mice could also have effects with age. The analysis of 1-year-old *Shroom3* heterozygous null mice exhibit glomerulosclerosis and smaller podocytes with foot process flattening and effacement. These mice exhibited mild albuminuria and increased protein-to-creatinine ratio that was not observed in controls indicating an age-dependent phenotype with reduced Shroom3 expression.^35^ Although these phenotypes could be explained by the fact that *Shroom3* heterozygous null mice exhibit reduced nephron number, similar hallmarks of glomerulosclerosis (podocyte loss, increased glomerular area, and mesangial expansion) were observed in 1-year-old *Shroom3* knockdown mice, that were generated using RNA interference (RNAi). As these hallmarks were not observed in control or young mice, this raises questions about the implication of reduced Shroom3 in the regulation of podocyte loss, glomerular volume, and glomerulosclerosis.^64,65^ The podocyte specific knockdown of *Shroom3* resulted in foot process effacement, albuminuria, and reduced glomerular volume without a loss of podocytes. In addition, unilateral nephrectomy in *Shroom3* knockdown mice resulted in reduced kidney mass, reduced glomerular and podocyte volume, but no changes in podocyte number. This study demonstrated that *Shroom3* knockdown results in the activation of AMP-activated protein kinase (AMPK), which diminishes protein synthesis, leading to reduced cellular content, lower glomerular and podocyte volume, and prevents the loss of podocytes.^66^ These protective effects were also seen in a congenic rat strain which showed that reincorporating the wild-type *Shroom3* gene resulted in reduced albuminuria and glomerulosclerosis with improved podocyte foot process effacement.^58^ RNAseq analysis of glomeruli confirms the protective role of Shroom3, as reduced Shroom3 expression resulted in a downregulation of numerous genes and signaling pathways involved in podocyte cytoskeletal organization.^35,58^

### Future Directions

Fundamental science has provided experimental evidence that supports GWAS studies and identified mechanisms that contribute to kidney disease. The phenotypes and age-dependent effects following the loss of Shroom3 offer opportunity to understand the interactions and functions of SHROOM3 in kidney development and its involvement in childhood and adult diseases.^56^ As SHROOM3 is expressed in the nephron progenitor cells, studies focused on understanding its role in early nephron formation and patterning will be especially valuable as there is a 10-fold variation in nephron number at birth, a 50% reduction in nephron number with aging, and native nephrons display limited regeneration capacity after injury.^67,68^ These characteristics indicate that there are major contributing factors during development and maintenance of postnatal nephrons that are not yet understood.^68^ During the initial stages of nephron formation, the nephron progenitor cells are highly dynamic, motile, generate membrane specializations, change their shape, and undergo mesenchyme-to-epithelial transition.^69,70^ Each of these processes requires dynamic actin rearrangement, which is dependent on Shroom3.^71,72^ A recent study has suggested that Shroom3 can exert nonautonomous effects in addition to the typical autonomous effects seen on neighboring cells to coordinate apical constriction during the closing *Xenopus* neural tube.^73^ This is an interesting area of study that can provide further insight into Shroom3 and its mechanisms of action.

Studies investigating neural tube closure demonstrate that Shroom3 works in a protein complex termed WERDS (Wnt4, EphrinB2, Ror2, Dishevelled [Dsh2], and SHROOM3).^74^ As Wnt4 is critical for nephron formation, it would be interesting to determine if Wnt4 and SHROOM3 work in a similar fashion in nephron progenitor cells.^75,76^ Studies have also demonstrated that SHROOM3 has binding sites for 14-3-3 which is a regulator of the Hippo signaling pathway.^18^ The Hippo signaling pathway is involved in the control of organ size, cell proliferation, and cell fate determination. Interestingly, downstream signaling molecules; LATS1/2, have been shown to control the epithelialization of nephron progenitor cells, indicating that SHROOM3 may interact with the Hippo signaling pathway during kidney development.^77^ In addition, the coordinated movement of epithelial cells, termed planar cell polarity (PCP), guides many processes important for kidney development, including branching morphogenesis and nephrogenesis, establishing epithelial polarity, and collective cell movements.^78^ Therefore, it is not surprising that SHROOM3 has been shown to bind to Dvl2, Vangl2, and Wnt5a, which are essential components of the PCP pathway in other organ systems.^39,79^ However, further investigation is needed to uncover the role of SHROOM3 and the regulation of these potential pathways, which could contribute to our understanding of how SHROOM3 influences kidney disease and development.

The many changes in epithelial morphology during kidney development occur due to actomyosin rearrangement, a primary role for SHROOM3. One of these morphological changes is apical constriction, which is crucial in the establishment of the developing tubular and glomerular epithelium.^26,35^ An essential step for SHROOM3 in the initiation of apical constriction is the recruitment and accumulation of F-actin and myosin II to the apical surface of developing cells.^6,7,20^ Studies have shown that this accumulation relies not only on SHROOM3, but its interactions with different binding partners. Some of these binding partners include vasodilator-stimulated phosphoprotein (Vasp) and Wiskott-Aldrich Syndrome protein (WASP), protein-coding gene paired-like homeodomain 2 (Pitx2), and N-cadherin.^80-85^ It is likely that these different binding partners initiate apical constriction in a cell-specific and stage-specific manner during kidney development.

## Conclusions

Although Shroom3 was originally implicated in the neural tube, its critical role in neural tube closure suggests that reductions in SHROOM3 expression and function in humans would have significant pathological effects in other organ systems. *SHROOM3* was first identified as a gene associated with kidney disease through GWAS, whereas the molecular biology defining the cellular mechanisms was lacking. However, experimental evidence in animal models offering insight into potential disease associations has uncovered how subtle alterations in SHROOM3 expression can be both pathological toward kidney disease and beneficial by exerting protective effects in the kidney. In each case, experimental evidence shows the role of SHROOM3 being established in various signaling pathways in many developing and postnatal model systems and is providing a more thorough understanding of the normal and pathological roles for SHROOM3 in kidney disease and beyond.

## Supplemental Material

sj-jpg-1-cjk-10.1177_20543581231212038 – Supplemental material for The Good and the Bad of SHROOM3 in Kidney Development and Disease: A Narrative Review Click here for additional data file. Supplemental material, sj-jpg-1-cjk-10.1177_20543581231212038 for The Good and the Bad of SHROOM3 in Kidney Development and Disease: A Narrative Review by Amy Paul, Allison Lawlor, Kristina Cunanan, Pukhraj S. Gaheer, Aditya Kalra, Melody Napoleone, Matthew B. Lanktree and Darren Bridgewater in Canadian Journal of Kidney Health and Disease
