# Supplementary figures and images for: The Good and the Bad of SHROOM3 in Kidney Development and Disease: A Narrative Review
Source: Can J Kidney Health Dis. 2023 Dec 13;10:20543581231212038. doi: 10.1177/20543581231212038 (PMC10722951; doi:10.1177/20543581231212038)

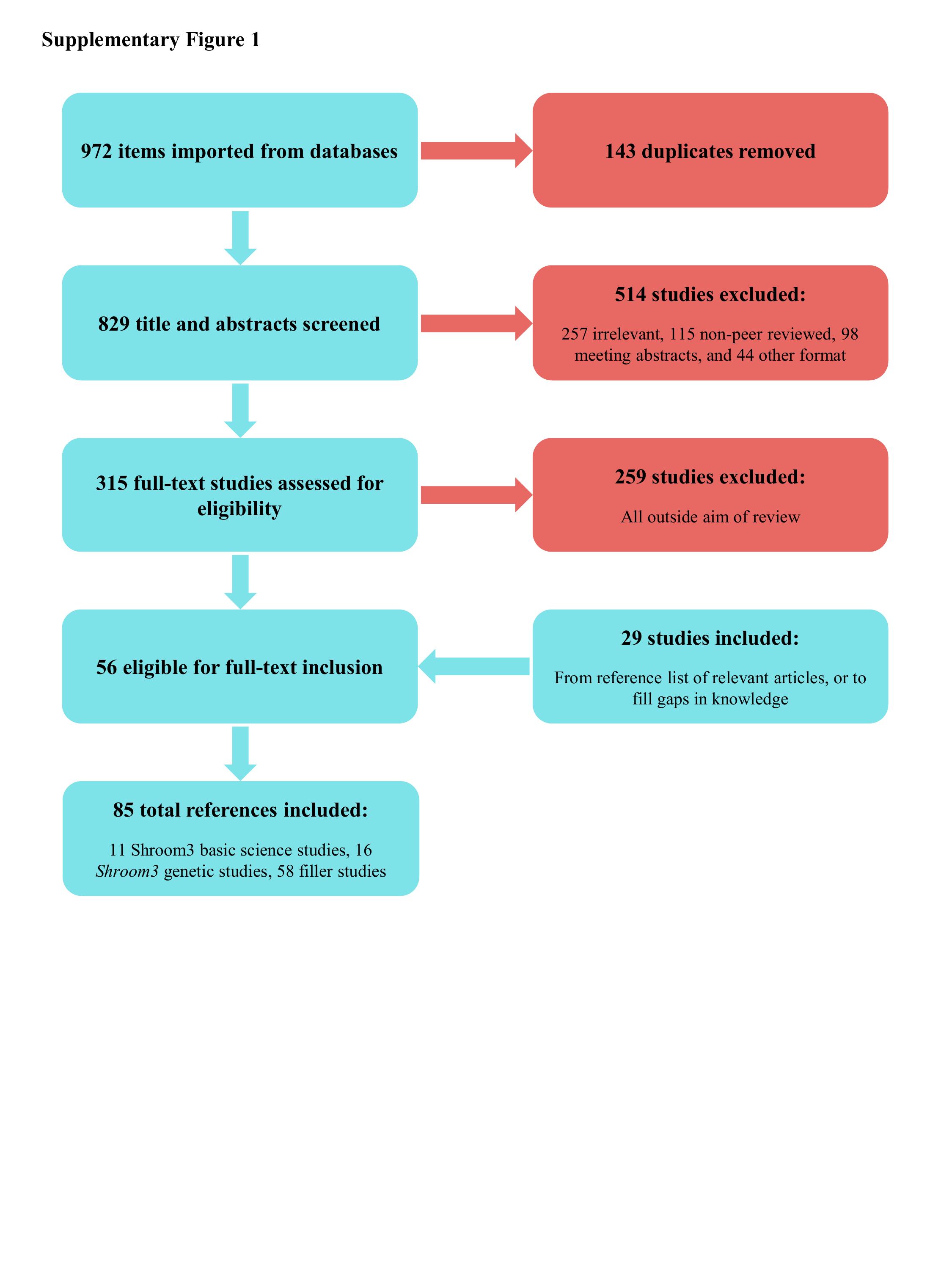

Supplement: sj-jpg-1-cjk-10.1177_20543581231212038 – Supplemental material for The Good and the Bad of SHROOM3 in Kidney Development and Disease: A Narrative Review [file sj-jpg-1-cjk-10.1177_20543581231212038.jpg]
